# Neutrophil Extracellular Traps Regulate HMGB1 Translocation and Kupffer Cell M1 Polarization During Acute Liver Transplantation Rejection

**DOI:** 10.3389/fimmu.2022.823511

**Published:** 2022-05-06

**Authors:** Yanyao Liu, Xingyu Pu, Xiaoyan Qin, Junhua Gong, Zuotian Huang, Yunhai Luo, Tong Mou, Baoyong Zhou, Ai Shen, Zhongjun Wu

**Affiliations:** ^1^ Department of Hepatobiliary Surgery, The First Affiliated Hospital of Chongqing Medical University, Chongqing, China; ^2^ Department of Liver Surgery and Liver Transplantation Center, West China Hospital of Sichuan University, Chengdu, China; ^3^ Department of General Surgery and Trauma Surgery, Children’s Hospital of Chongqing Medical University, National Clinical Research Center for Children Health and Disorders, Ministry of Education Key Laboratory of Child Development and Disorders, National Clinical Research Center for Children Health and Disorders, China International Science and Technology Cooperation Base of Child Development and Critical Disorders, Chongqing Key Laboratory of Pediatrics, Chongqing, China; ^4^ Department of Hepatobiliary Pancreatic Tumor Center, Chongqing University Cancer Hospital, Chongqing, China

**Keywords:** neutrophil extracellular traps (NETs), high mobility group box-1 (HMGB1), liver transplantation, Kupffer cell, acute rejection

## Abstract

Neutrophil extracellular traps (NETs) play important roles in hepatic ischemic reperfusion injury (IRI) and acute rejection (AR)-induced immune responses to inflammation. After liver transplantation, HMGB1, an inflammatory mediator, contributes to the development of AR. Even though studies have found that HMGB1 can promote NET formation, the correlation between NETs and HMGB1 in the development of AR following liver transplantation has not been elucidated. In this study, levels of serum NETs were significantly elevated in patients after liver transplantation. Moreover, we found that circulating levels of NETs were negatively correlated with liver function. In addition, liver transplantation and elevated extracellular HMGB1 promoted NET formation. The HMGB1/TLR-4/MAPK signaling pathway, which is initiated by HMGB1, participates in NET processes. Moreover, in the liver, Kupffer cells were found to be the main cells secreting HMGB1. NETs induced Kupffer cell M1 polarization and decreased the intracellular translocation of HMGB1 by inhibiting DNase-1. Additionally, co-treatment with TAK-242 (a TLR-4 inhibitor) and rapamycin more effectively alleviated the damaging effects of AR following liver transplantation than either drug alone.

## Introduction

Orthotopic liver transplantation is the only effective therapy for acute liver failure and end-stage liver disease (1, 2). Immunotherapies significantly attenuate the acute rejection (AR) of liver allografts, but they also cause a series of complications including drug-induced liver injury, tumor recurrence, and severe infection (3, 4). Therefore, novel therapeutic strategies for alleviating the severity of acute rejection and minimization or eliminating immunosuppression are required. Neutrophils are critical in the pathophysiology of both ischemia–reperfusion injury (IRI) and AR of liver transplantation. They are the major amplifiers of hepatic IRI as well as the AR associated with liver transplantation (5–7).

Neutrophil extracellular traps (NETs) are net-like structures formed by neutrophils. They are composed of nuclear DNA studded with granule proteins, histones, and cytoplasmic antimicrobials (8, 9). Although a previous study established that NETs have a role in a variety of liver diseases, including liver IRI, alcohol-induced liver disease, and hepatocellular carcinoma, the mechanism by which NETs cause AR following liver transplantation requires additional investigation (10, 11).

Toll-like receptor 4 (TLR-4) is expressed by a variety of immune-related cells in the liver (12). TLR-4-mediated innate immune responses are triggered upon AR of liver transplantation and result in the release of inflammatory cytokines *via* activation of MAPK signaling, followed by induction of liver inflammation (13). Therefore, the endogenous TLR-4 ligand promotes AR-associated liver inflammatory responses (14).

Following liver transplantation, high-mobility group box-1 (HMGB1), an AR activator after liver transplantation, can activate an immune response (15). Serum HMGB1 levels are significantly elevated in liver transplant patients, and HMGB1 overexpression has been associated with AR development following liver transplantation (16, 17). However, the cells responsible for the majority of HMGB1 secretion in the liver during immune-mediated liver injury and the stimuli that activate HMGB1 intracellular translocation to induce AR following liver transplantation remain unknown. Intriguingly, HMGB1 has been shown to stimulate the production of NETs in liver IRI, indicating that HMGB1 is a critical TLR-4 endogenous ligand involved in hepatic ischemia/reperfusion injury (18–20). Although previous research has demonstrated that HMGB1 induces NETosis and liver ischemia/reperfusion injury in a TLR-4-dependent manner, the specific mechanism by which HMGB1 induces NET formation in patients with AR following liver transplantation is unknown.

To investigate neutrophil ability to undergo NETosis, we used patients with liver transplantation and liver transplantation rat models. Additionally, the pathways that trigger HMGB1-induced NETosis were studied, and it was discovered that NETs promote Kupffer cell M1 polarization and HMGB1 intracellular translocation, aggravating AR following liver transplantation. Finally, we emphasize the importance of combining TAK-242 (a TLR-4 inhibitor) and rapamycin (an mTOR inhibitor) treatment in patients with AR following liver transplantation.

## Materials and Methods

### Ethical Statement

This study was approved by the Ethics Committee of Animal and Human Experimentation of Chongqing Medical University. All human subjects signed informed consent for participation in this study, and all experiments were performed in accordance with the Declaration of Helsinki guidelines. Efforts were made to minimize animal distress and pain, and we used a small number of animals based on the 3Rs principle.

### Human Blood Samples

Peripheral blood samples were obtained from 13 liver transplant recipients. Liver transplantation recipients were handled according to institutional protocols by experienced transplant clinicians. Blood tests such as liver function tests, full blood examination, and serum immunosuppressant levels were performed regularly to monitor the clinical course. Additionally, 10 ml of blood was on post-transplant days of 1, 3, 7, 14, and 28 for NET investigation.

### Human Primary Neutrophils and Serum

Samples were obtained from antecubital veins in vacuum blood collection tubes. Serum was centrifuged at 2,000×g for 10 min, within 1 h of collection, aliquoted, and stored at -80°C for assaying. Human peripheral blood neutrophils were obtained using previously reported Ficoll-Dextran techniques, and purified neutrophils (>95%) were analyzed using flow cytometry and manual counting for CD11b and Ly6G expression. Trypan blue was used to determine the viability of neutrophils (>95%).

### Isolation of Primary Kupffer Cells and Hepatocytes

Primary Kupffer cells were isolated from liver tissues and purified using modified methods, which consist primarily of three steps: *in situ* liver perfusion with collagenase digestion, gradient centrifugation, and selective adherence. Primary hepatocyte isolation *in situ* liver perfusion was carried out in the same manner as primary Kupffer cell isolation. Centrifugation of the liver homogenate was performed three times at 50 × g (4°C) for 3 min. Cell pellets were seeded onto plates coated with rat tail collagen and cultured in RPMI 1640 Medium (HyClone, Logan, UT, USA) supplemented with 15% fetal bovine serum (FBS; HyClone, USA) and 1% streptomycin/penicillin (Sigma Aldrich, St. Louis, MO, USA).

### HMGB1 Inhibition

Glycyrrhizin is a potent inhibitor of HMGB1. Glycyrrhizin can bind directly to HMGB1 and does not affect other chemokines. After 24 h of treatment with glycyrrhizin (15 mg/kg), rats were treated with LPS.

### Separation of Cytoplasmic and Nuclear Extracts

Cell nuclei and cytoplasms were separated using a cell fractionation kit (Cell Signaling Technology, Shanghai, China). Trypsinization was performed on approximately 5 × 10^6^ cells. Cells were spun down at 350 × g for 5 min. The pellets were then resuspended in 500 μl of cytoplasm isolation buffer, vortexed for 5 s, incubated on ice for 5 min, and then centrifuged again for 5 min at 500 × g. The supernatant was the cytoplasmic fraction. The pellets were resuspended in 500 μl of membrane isolation buffer, vortexed for 15 s, incubated on ice for 5 min, and centrifuged at 8,000 × g for 5 min. The supernatant was the membrane and organelle fraction. The pellets were then resuspended in 250 μl of nucleus isolation buffer. Three times, the samples were sonicated for 5 s at 15% power. This was the nuclear fraction.

### Neutrophil Stimulation

Neutrophils (5 × 10^6^) were cultured in 200 µl of DMEM supplemented with HMGB1 (5, 10, 20, or 40 ng/ml; Beyotime, Shanghai, China) or phorbol 12-myristate 13-acetate (100 nm; PMA; Beyotime, Shanghai, China) in 48-well plates. The cells were incubated at 37°C for 120 min in a 5% CO_2_ environment. Neutrophil pretreatment was performed using TAK-242 (10 µM; MedChemExpress, Princeton, NJ, USA) or LPS (25 µg/ml; Beyotime, Shanghai, China) 30 min before stimulation.

### Isolation of NETs and Kupffer Cell Stimulation

To stimulate neutrophils, PMA (100 nm; Beyotime, Shanghai, China) was supplemented to the media after which 4 h of incubation in a 5% CO_2_ environment at 37°C was done. The medium was then gently aspirated and replaced with new precooling PBS (calcium and magnesium-free), and the remaining adhesions were collected by scrapings. The collected cells and NETs were transferred to a 15-ml conical tube followed by 10 min of centrifugation (400 g) at 4°C. Supernatants containing NETs were obtained and centrifuged (1,800 g) for 10 min at 4°C to precipitate DNA. Supernatants were discarded, and the remaining pellets were resuspended in precooling PBS. Subsequently, the DNA concentration of every sample was determined by spectrophotometry.

### Animal Liver Transplantation Models

Male inbred Brown Norway rats (BN) and Lewis rats (LEW) (SPF grade, 220–250 g) were obtained from the Chongqing Medical University experimental animal center (Chongqing, China). The rats were maintained in a pathogen-free environment and randomly assigned to 5 groups of 12 rats each. A novel magnetic anastomosis technique was used to conduct orthotopic liver transplantation, as reported by Yang (21). An abdominal incision was made in the sham group rats to expose the hepatic portal vein. After liver transplantation, rats in the liver transplantation (LT) group received no treatment (LEW rats were donors, BN rats were recipients). TAK-242 (1.5 mg/kg/day; MedChemExpress, NJ, USA) was administered intraperitoneally to rats in the LT+TAK-242 group following liver transplantation. Rats in the LT+rapamycin group underwent liver transplantation followed by rapamycin (1 mg/kg/day; MedChemExpress, NJ, USA) tail vein injection. Rats in the LT+TAK-242+rapamycin group underwent liver transplantation followed by intraperitoneal administration of TAK-242 (1.5 mg/kg/day) and tail vein rapamycin (1 mg/kg/day) injection. After orthotopic liver transplantation, rats were sacrificed at different time points, and liver tissues, as well as serums, were collected and stored at ˗80°C.

### Hepatic Aminotransferase Analysis

The serum levels of alanine aminotransferase (ALT), total bilirubin (TBIL), and aspartate aminotransferase (AST) were assessed using liver enzyme kits (Jiancheng Bioengineering Institute, Nanjing, China) according to the manufacturer’s instructions.

### Quantification of Extracellular DNA

Extracellular DNA was measured using the Quant-iT PicoGreen dsDNA Assay Kit (Life Technologies, Grand Island, NY, USA) to evaluate NET formation according to the product guide. Each sample was analyzed three times.

### ELISA

Serum neutrophil elastase (NE) concentrations in human peripheral blood were determined using ELISA kits (JYM, Wuhan, China) according to the manufacturer’s instructions.

### qRT-PCR

Rat liver tissues were collected, and total RNA was extracted using the TRIzol Reagent (Takara, Tokyo, Japan). cDNA was synthesized using the PrimeScript RT Reagent (Takara, Tokyo, Japan) according to the manufacturer’s instructions. Primers (**Table 1**) for qPCR amplification were procured from Sangon Biotech (Sangon Biotech, Shanghai, China). Experiments were performed in triplicate, and data analysis was performed using the 2-ΔΔCT method.

**Table 1:** Primer sequences.

Name	Forward primer	Reverse primer
TNF-α	CTACGTGCTCCTCACCCACACCGT	ACCTCAGCGCTGAGCAGGTCCCCC
IL-1β	AGGGCTGCTTCCAAACCTTTGACC	ACTGCCTGCCTGAAGCTCTTGTTG
IL-6	CTGATTGTATGAACAGCGATGATG	AACTCCAGAAGACCAGAGCAGATT
CXCL1	GGGGCGCCCGTCGCCAATGAGCTG	TCACCTTCAAACTCTGGATGTTCT
CXCL2	GTCCTGCTCCTCCTGCTGGCCACC	GTCGTCAGGCATTGACAGCGCAGC
Ly6G	TGTGCAGAAAGAGCTCAGGGGCTGG	AGTGGGGCAGATGGGAAGGCAGAGA
GADPH	GGTGGACCTCATGGCCTACA	CTCTCTTGCTCTCAGTATCCTTGCT

### Western Blotting

Total liver proteins were extracted by lysing them in a radioimmunoprecipitation assay buffer supplemented with a proteinase inhibitor cocktail. Then, 10–20 µg of the extracted protein samples was run on SDS-PAGE gels (10%) and transferred to polyvinylidene fluoride membranes. The membranes were incubated overnight at 4°C with primary antibodies (**Table 2**) followed by 1 h at room temperature with horseradish peroxidase-conjugated secondary antibody (Amersham Biosciences, Amersham, UK). A gel imaging system (ChemiScope 2850, Clinx Science, Shanghai, China) detected the signal *via* a chemiluminescent reaction. ImageJ was used to quantify immunoreactive bands.

**Table 2:** Antibody for immunofluorescence staining and Western blot.

Primary antibody	Dilution	Supplier	Code
Erk1/2	WB:1/1000	CST	4695
p-Erk1/2	WB:1/1000	CST	4370
P38	WB:1/1000	CST	8690
p-p38	WB:1/1000	CST	4511
JNK	WB:1/1000	CST	9252
p-JNK	WB:1/1000	CST	4668
iNOS	IF:1/500 WB:1/1000	Proteintech	18985-1-AP
TNF-α	WB:1/1000	Proteintech	17590-1-AP
IL-6	WB:1/1000	Proteintech	66146-1-Ig
IL-1β	WB:1/1000	Proteintech	16806-1-AP
TLR-4	WB:1/300	Abcam	ab217274
H3cit	IF:1/500 WB:1/1000	Abcam	ab5103
Ly6G	IF:1/500	Santa Cruz	Sc-53515
MPO	IF:1/100	Abcam	ab208670
NE	IF:1/70	Abcam	ab131260
PR3	IF:1/100	Abcam	ab270441
NOX2	WB:1/1000	Abcam	ab129068
NOX4	WB:1/1000	Abcam	ab133303
PAD4	WB:1/1000	Abcam	ab214810
HMGB1	WB:1/1000	CST	6893
Lamin B1	WB:1/1000	CST	13435S
β-actin	WB:1/1000	CST	4970

### Immunofluorescence Analysis

To detect neutrophil-associated enzymes in NETs, primary antibodies against neutrophil elastase (NE), citrullinated histone-3 (H3Cit), myeloperoxidase (MPO), and proteinase 3 (PR3) were utilized. Lymphocyte antigen 6G (Ly6G) was used as the primary antibody to determine neutrophil infiltration levels and accumulation in the liver. H3cit and MPO were used as the primary antibodies to determine the NET formation in the liver. DAPI was used to stain the DNA/NETs. Cells were incubated for 3 min at room temperature in the presence of a 1:1,000 dilution of DAPI. Then, cells were washed twice using PBS and covered with an anti-fluorescence quencher. Inducible nitric oxide synthase (iNOS) was used as the primary antibody to determine M1 polarization levels of Kupffer cells under different conditions. A fluorescence microscope or confocal fluorescence microscope was used to determine the structure and location of NETs.

### Hematoxylin–Eosin Staining and Terminal Deoxynucleotidyl Transferase dUTP Nick-End Labeling

The grafts were fixed in 4% paraformaldehyde for 24 h. After embedding, tissues were sectioned (5 µM) and stained with hematoxylin–eosin (HE). The Banff criteria were used for scoring AR. The terminal deoxynucleotidyl transferase dUTP nick end labeling (TUNEL) kit (Beyotime, Shanghai, China) was used to determine the level of hepatic apoptosis according to the manufacturer’s instructions. At different magnifications, a fluorescent microscope was used for imaging.

### Statistical analysis

The data were presented as mean ± SE for n = 3. Student’s t-tests, one-way ANOVA, Mann–Whitney U test, and chi-square test were conducted using GraphPad Prism 8.0 and SPSS 22.0. P *<* 0.05 was considered statistically significant.

## Results

### Orthotropic Liver Transplantation in Rats Induces a Remarkable Acute Rejection Response

**Figure 1:**
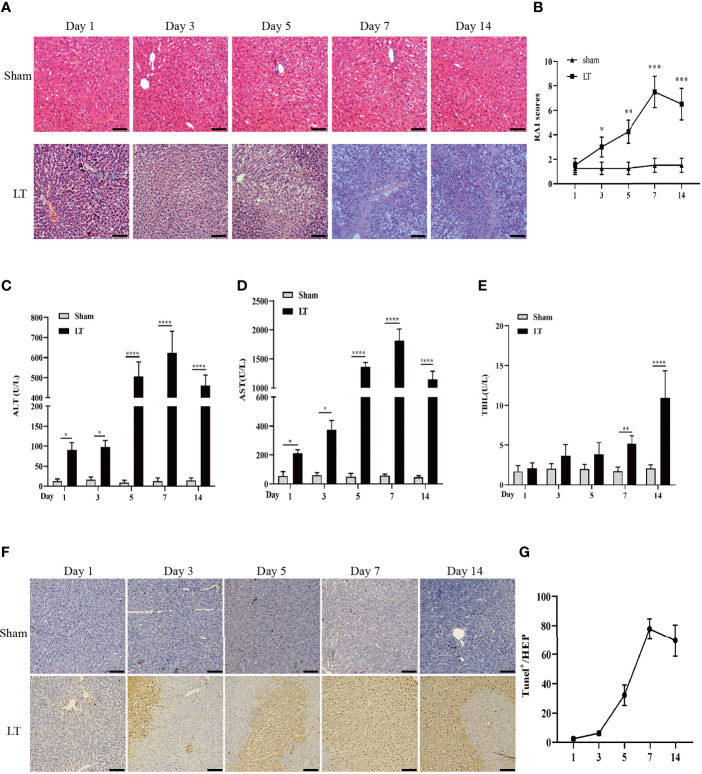
AR responses in isograft and allograft groups. **(A)** Hepatic pathologic alteration following liver transplantation on days 1, 3, 5, 7, and 14 (magnification, ×200; scale bar = 100 μm). **(B)** Histological classification was determined by RAI based on Banff’s scheme. **(C–E)** Serum ALT, TBIL, and AST levels on days 1, 3, 5, 7, and 14 following liver transplantation. **(F, G)** Hepatic apoptosis detected by TUNEL following liver transplantation on days 1, 3, 5, 7, and 14 (magnification, ×200; scale bar = 100 μm). All differences denoted by asterisks were subjected to Student’s t-tests. The values are presented as mean ± SD (n = 6). ^*^ *P* < 0.05, ^**^ *P* < 0.01, ***P < 0.001, ^****^ *P* < 0.0001.

HE staining revealed a severe, progressive AR response in the liver tissues of rats in the LT group, which was characterized by bile duct damage, serious hepatocyte necrosis, and leucocyte infiltration and peaked on day 7 following transplantation (**Figure 1A**). On day 7 following liver transplantation, RAI scores were significantly elevated in the liver transplantation group than in the other groups (**Figure 1B**). The serum parameters for both groups were assessed using a microplate assay. ALT and AST levels were observed to be elevated postoperatively and peaked at day 7. Additionally, TBIL levels were elevated and peaked on the 14th day (**Figure 1C–E**). Similarly, hepatic apoptosis was significantly increased and peaked within 7 days post-transplantation (**Figures 1F, G**).

### Neutrophil Infiltration and NET Deposition in the Liver Cause Acute Inflammation in the Livers of Rat Models

**Figure 2:**
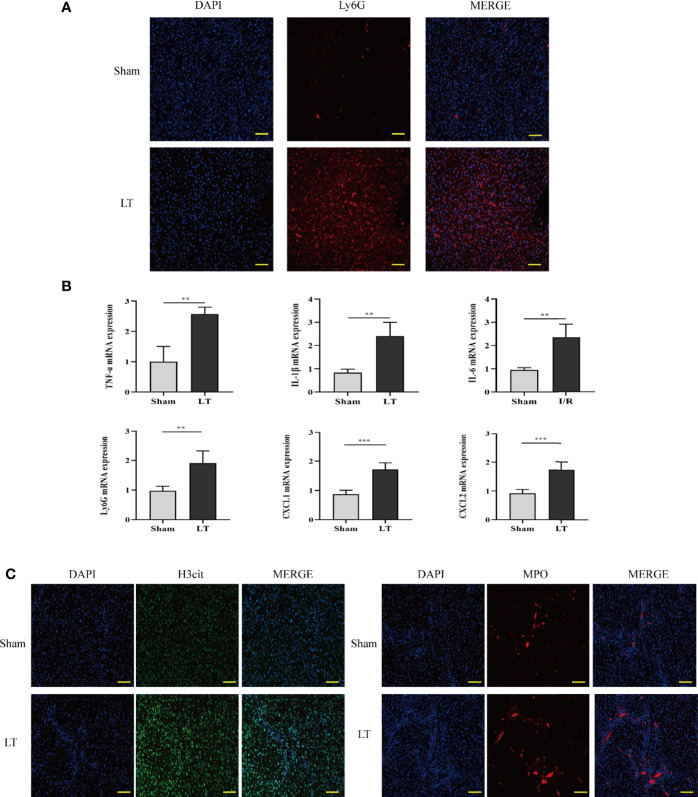
Neutrophil infiltration and NET deposits in the liver induce an inflammatory response in the AR of liver transplantation rats. **(A)** Immunofluorescence assessment of the neutrophil marker (Ly6G) for labeling neutrophil locations in livers (magnification, ×200; scale bar = 100 μm). **(B)** mRNA TNF-α, IL-6, Ly6G, IL-1β, CXCL1, and CXCL2 levels in liver tissues. **(C)** Representative immunofluorescence micrographs demonstrating the co-localization of NET components, including H3Cit, DAPI, and MPO in the livers of liver transplantation rats (magnification, ×200; scale bar = 100 μm). Data were analyzed by the Mann–Whitney test. Data are presented as mean ± SD (n = 6). ^**^ *P* < 0.01, ^***^ *P* < 0.001.

To investigate the presence of neutrophils in the AR of liver transplantation models, we used the specific neutrophil marker Ly6G to identify neutrophils in the liver. The LT group rats had a higher number of neutrophils than the sham group. Additionally, Ly6G mRNA levels were significantly increased in the LT group (**Figures 2A, B**). To establish a connection between neutrophil infiltration and liver inflammation, qRT-PCR was conducted to determine the expression levels of inflammatory and adhesion cytokines such as TNF-α, CXCL1, IL-1β, CXCL2, and IL-6. In samples from the LT group, there was a significant increase in pro-inflammatory and adhesion cytokines (**Figure 2B**). Then, we determined whether the infiltrated neutrophils contributed to the NET formation in the liver from the AR of liver transplantation models. Typical NETs were labeled using MPO and H3Cit markers. In comparison to the sham group, typical NET structures were found in the livers of the LT group (**Figure 2C**).

### Circulating Serum NETosis Products Are Elevated in Liver Transplant Patients

**Figure 3:**
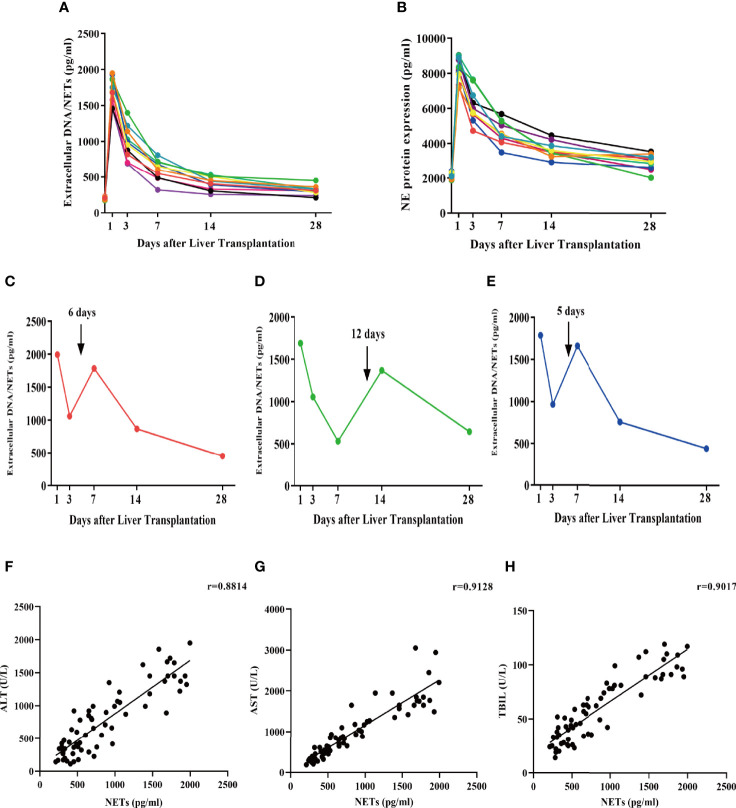
Serum extracellular NETs and circulating NETosis products are increased in patients undergoing liver transplantation. **(A, B)** Extracellular NET and NET levels (NE) were determined in the serum of patients undergoing liver transplantation without AR at days 1, 3, 7, 14, and 28. **(C–E)** Extracellular NETs were measured in the serum of liver transplantation patients with AR at days 1, 3, 7, 14, and 28. **(F–H)** Pearson correlation analysis of NETs with ALT, AST, and TBIL.

To determine whether liver transplantation initiates NET formation, we measured serum levels of extracellular DNA/NETs as well as NETosis product NE in patients without any complications before the operation and on days 1, 3, 5, 7, and 14 following liver transplantation (**Figures 3A, B**). The demographics and clinical characteristics of all patients are shown in **Table 3**. NET formation was enhanced, and a stereotypic decline occurred with extended recovery time after liver transplantation. Three participants with AR who required treatment (baseline characteristics are summarized in **Table 3**) had abnormally elevated extracellular DNA/NETs following diagnosis of AR. Extracellular DNA/NETs decreased following treatment (oral rapamycin twice daily) compared to pretreatment (**Figures 3C–E**). Additionally, serum levels of extracellular DNA/NETs and liver function markers (ALT, AST, TBIL) were determined in patients without any complications before the operation and on days 1, 3, 5, 7, and 14 following liver transplantation. Pearson correlation analysis was performed to determine the correlation between NETs and traditional liver function (ALT, AST, TBIL) markers. The data indicated that circulating levels of NETs were positively correlated with ALT, AST, and TBIL in patients undergoing liver transplantation (**Figures 3F–H**).

**Table 3:** Characteristics of the base line.

	Overall (n = 13)	Uneventful (n = 10)	tBPAR (n = 3)
	Mean or count (SD or %)	Mean or count (SD or %)	Mean or count (SD or %)
Recipient demographics			
Recipient age (y)	48.1 (8.1)	48.8 (7.7)	46.0 (11.1)
Recipient sex			
Male	10 (76.9%)	8 (80%)	2 (66.7%)
Female	3 (23.1%)	2 (20%)	1 (33.3%)
Cause			
Cryptogenic	3 (23.1%)	3 (30%)	0 (0%)
Alcohol	2 (15.4%)	1 (10%)	1 (33.3%)
Viral hepatitis	1 (7.7%)	1(10%)	0(0%)
HCC	7 (53.8%)	5 (50%)	2 (66.7%)
Donor demographics			
Donor age (y)	35.5 (5.5)	37.3 (7.7)	50.7 (4.0)
Donor sex			
Male	11 (84.6%)	8 (80%)	3 (100%)
Female	2 (15.4%)	2 (20%)	0 (0%)
LT characteristics			
Operative time (min)	495 (84)	449 (81)	512 (109)
Warm ischemic time (min)	41 (6)	49 (6)	57 (3)
Cold ischemic time (min)	421 (51)	399 (30)	497 (25)
Maximum ALT (U/L)	2,307 (1018)	2,075 (900)	3,080 (1,187)
Hospital length of stay (day)	27 (6)	25 (4)	32 (11)
ICU length of stay (day)	6 (3)	5 (1)	10 (3)

ALT, alanine aminotransferase; HCC, hepatocellular carcinoma; LT, liver transplantation; SD, standard deviation; tBPAR, treated biopsy-proven acute rejection.

### Liver Transplantation Promotes NET Formation

**Figure 4:**
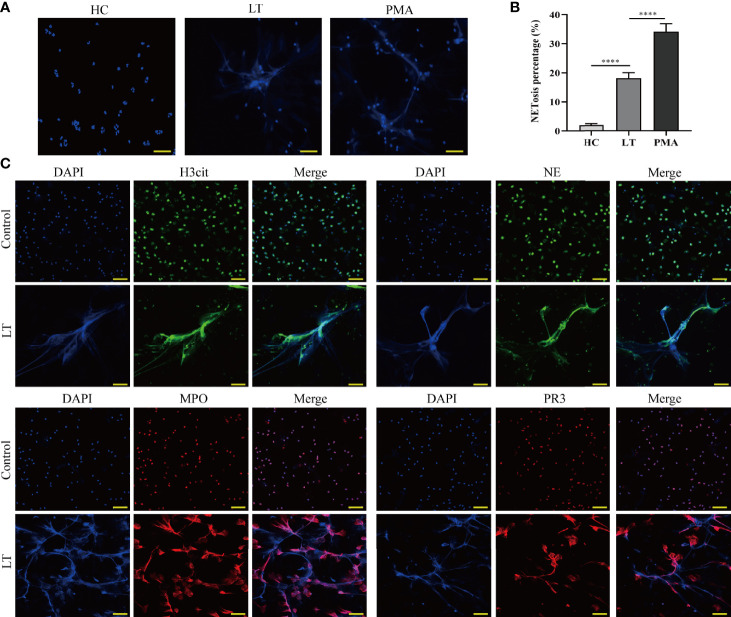
Release of NETs from peripheral blood neutrophils in liver transplantation patients. **(A)** Neutrophil cells were obtained from the peripheral venous blood of healthy controls (HC) and liver transplantation patients. They were incubated at 37°C for 2 h without stimulation. PMA was used as a positive control (magnification, ×200; scale bar = 100 μm). **(B)** Quantification of neutrophil NETosis. **(C)** Immunofluorescence assessment of NET formation as determined by colocalization of H3Cit, NE, MPO, PR3, and DAPI (magnification, ×200; scale bar = 100 μm). n = 6 for each group. Data were analyzed using the chi-square test. ****P < 0.0001.

To verify the presence of NET formation in transplanted individuals, we isolated neutrophils from patients receiving or not receiving liver transplantation (**Figure S1**). Neutrophils were examined to determine whether they could spontaneously form NETs, using PMA as a positive control. We discovered that whereas neutrophils from patients undergoing liver transplantation spontaneously formed NETs, neutrophils from healthy controls hardly formed NETs (**Figures 4A, B**). Following that, NET components H3Cit, NE, MPO, and PR3 were analyzed using confocal fluorescence microscopy. The results indicated that NET components colocalized with neutrophil extracellular chromatin fibers from liver transplantation patients but not with neutrophils from healthy controls (**Figure 4C**).

### HMGB1 Promote NET Formation

**Figure 5:**
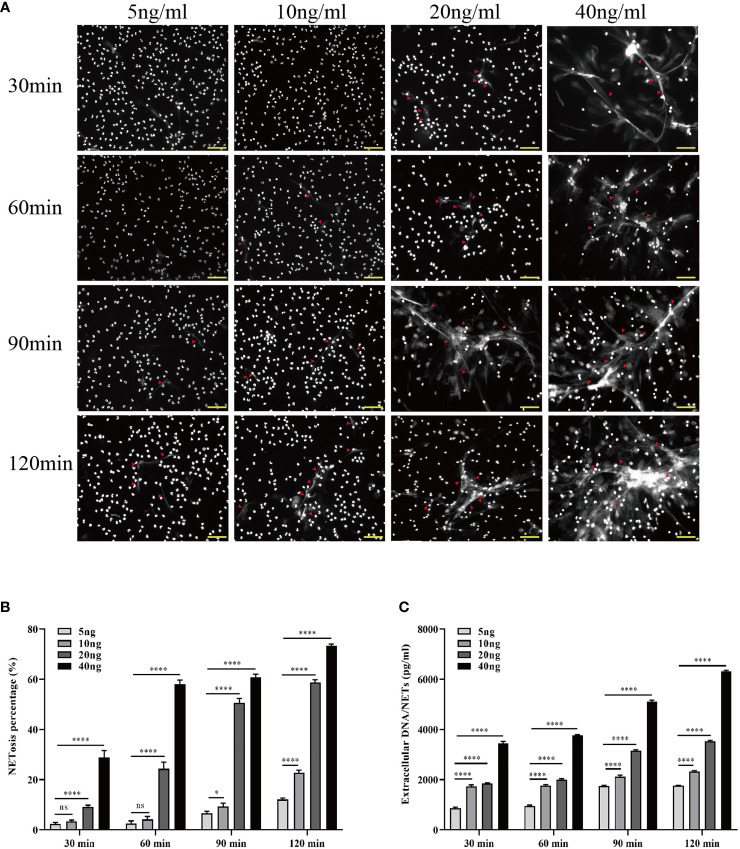
Increased HMGB1 induces more NETosis *in vitro*. **(A)** Induction of NETosis in neutrophils from healthy individuals using different concentrations of HMGB1 (5, 10, 20, 40 ng/ml) at various time points (30, 60, 90, 120 min). **(B)** Quantification of neutrophil NETosis percentage. **(C)** Extracellular DNA/NETs were quantified every 30 min over a period of 120 min. n = 6. Differences were determined using one-way ANOVA. ^ns^ *P* > 0.05, ^*^ *P* < 0.05, ^***^ *P* < 0.001, ^****^ *P* < 0.0001.

To investigate whether HMGB1 induces NET formation *in vitro*, neutrophils from 3 healthy individuals were isolated and cultured in DMEM supplemented with varying concentrations (5, 10, 20, and 40 ng/ml) of HMGB. The association between stimulation conditions and NET formation was determined at various time points (30, 60, 90, and 120 min). During the 2-h incubation with HMGB1 (40 ng/ml), the majority of nuclei decondensed, while the netting neutrophils continually formed with increasing time (**Figure 5A**). There was an elevated NETosis percentage (**Figure 5B**) and a NET level (**Figure 5C**) that was dependent on the duration of exposure to HMGB1 as well as the concentration.

### HMGB1 Induces NET Formation Through the TLR-4/MAPK Signaling Pathway

**Figure 6:**
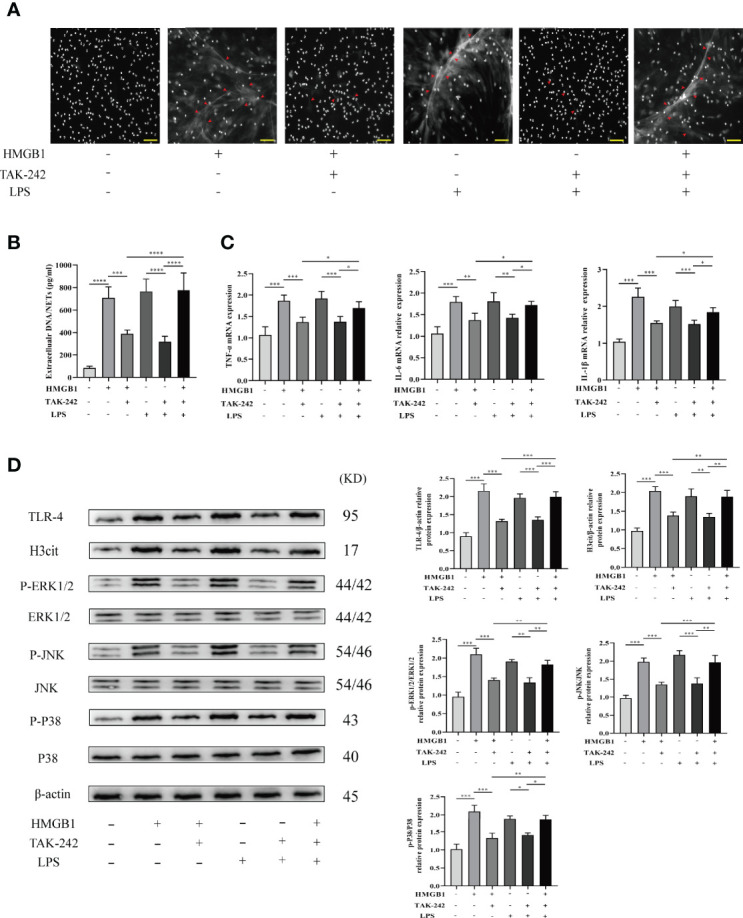
TAK-242 suppresses the MAPK signaling pathways and alleviates NET deposition in rat liver transplantation. **(A)** Following transplantation, NETs were stained with DAPI in the livers of rats (magnification, ×200; scale bar = 100 μm). **(B)** Quantification of extracellular DNA/NETs in different groups. **(C)** The mRNA level of IL-1β, TNF-α, and IL-6 in liver tissues from different groups. **(D)** Protein expression levels of MAPK signaling pathway factors. n = 6 for each group. ANOVA was used to determine the statistical differences between groups. ^*^ *P* < 0.05, ^**^ *P* < 0.01, ^***^ *P* < 0.001, ^****^ *P* < 0.0001.

LPS was utilized as a TLR-4 agonist to determine whether TAK-242 (TLR-4 inhibitor) inhibits NET formation induced by blockage of TLR-4 *in vitro*. Isolated neutrophils were divided into four groups including the no treatment group, pretreatment with HMGB1 (40 ng/ml) group, pretreatment with HMGB1 and TAK-242 (10 μg/ml) group, and pretreatment with HMGB1, TAK-242 (10μg/ml), and LPS (25 μg/ml) group. Representative fluorescent images are shown in **Figure 6A**. The percentage of NET formation was determined manually, while inflammatory gene (IL-6, TNF-α, and IL-1β) expression was determined using real-time PCR. Therefore, TAK-242 significantly suppressed NET formation and the expression of inflammatory factors induced by HMGB1, while LPS markedly reversed this suppressive effect of TAK-242 (**Figures 6B, C**). Western blot analysis was used to determine the expression levels of TLR-4/MAPK signaling pathway-related proteins. Consistent with previous findings, TAK-242 significantly inhibited the TLR-4/MAPK signaling pathway activation induced by HMGB1, while LPS significantly reversed this inhibitory effect (**Figure 6D**).

### Kupffer Cells Are the Main HMGB1 Secretory Cells in the Liver

**Figure 7:**
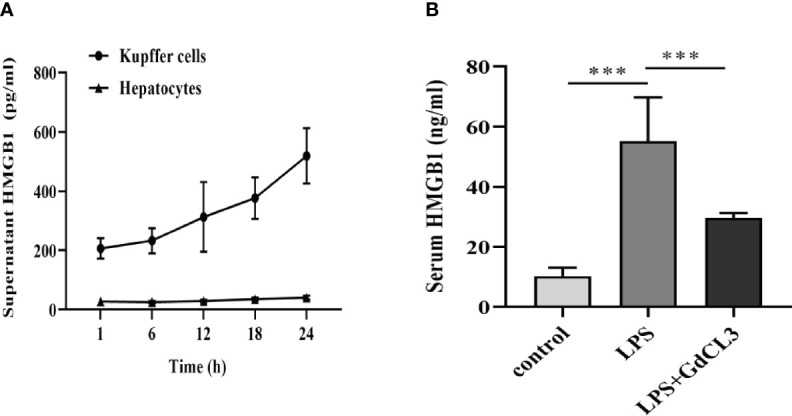
Kupffer cells are the major HMGB1-secreting cells. **(A)** ELISA analysis of supernatant HMGB1 levels in primary rat Kupffer cells and hepatocytes following stimulation by LPS (1 μg/ml). **(B)** Rats were intraperitoneally administered with LPS (8 mg/kg) in the absence or presence of GdCl3 (20 mg/kg for 24 h before LPS injection) injection. Serum HMGB1 was measured by ELISA, 12 h after the injection. n = 6 for each group. One-way ANOVA was used to determine the statistical differences between groups. ^***^ *P* < 0.001.

We investigated whether Kupffer cells or hepatocytes were the main HMGB1-secreting cells during immune-mediated liver injury. Primary Kupffer cells and hepatocytes were obtained from healthy rats and pretreated with LPS at different time durations (1, 6, 12, 18, and 24 h). *In vitro*, Kupffer cell supernatants contained significantly higher levels of HMGB1 than hepatocyte supernatants (**Figure 7A**). To illustrate the effect of Kupffer cell depletion on HMGB1 secretion, rats were pretreated with GdCL3 (a macrophage inhibitor). In comparison to the LPS-treated group, we observed that GdCL3 significantly suppressed the serum HMGB1 levels in the LPS+GdCL3 group *in vivo* (**Figure 7B**).

### NET Formation Stimulates Kupffer Cell M1 Polarization and Intracellular Translocation of HMGB1

**Figure 8:**
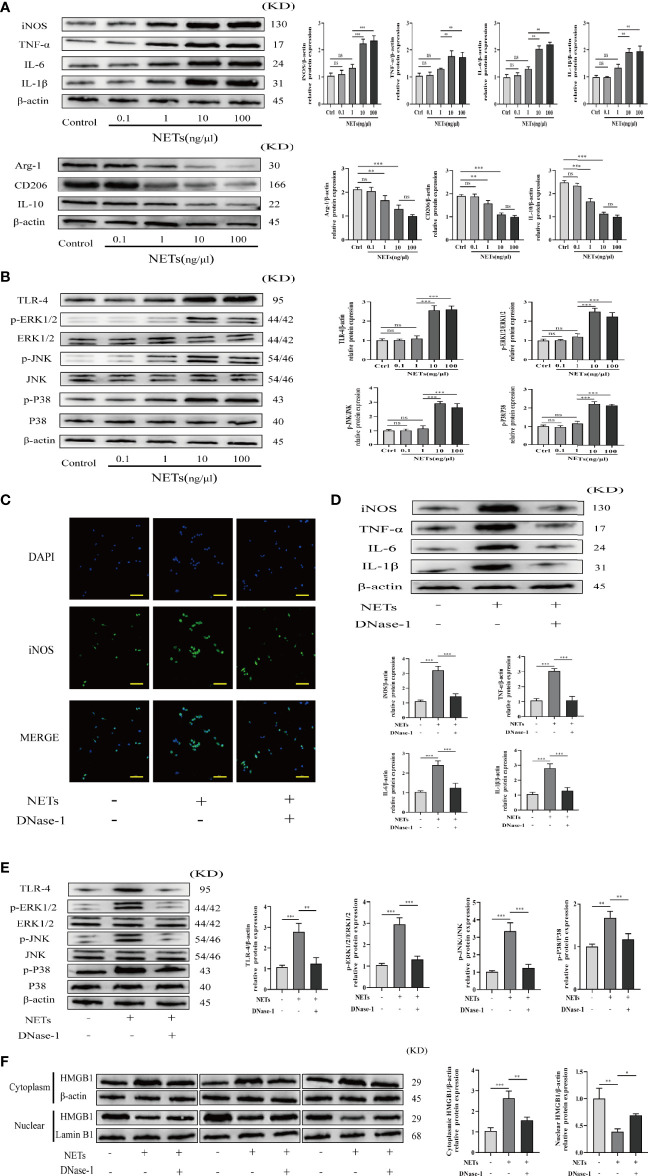
NETs promote M1 polarization of Kupffer cells and HMGB1 intracellular translocation. **(A, B)** Expression of M1/M2 polarization markers and TLR-4/MAPK signaling pathway factors in Kupffer cells induced by different concentrations of NETs. **(C)** Immunofluorescence assessment of Kupffer cell M1 polarization as determined by iNOS colocalization and DAPI (magnification, ×400; scale bar = 50 μm). **(D, E)** Expression of M1 polarization markers and TLR-4/MAPK signaling pathway factors in Kupffer cells stimulated by NETs in the absence or presence of DNase-1. **(F)** Immunoblotting was used to determine the translocation of HMGB1 from the nucleus to the cytoplasm. n = 6. One-way ANOVA was used to determine the statistical differences between groups. ^ns^ *P* < 0.05, ^*^ *P* < 0.05, ^**^ *P* < 0.01, ^***^ *P* < 0.001.

To confirm the function of NETs in promoting Kupffer cell polarization toward the M1 phenotype, we obtained primary Kupffer cells from healthy rats (**Figure S1**) and incubated them in DMEM with varying concentrations of NETs (0.1, 1, 10, 100 ng/μl). Western blot analysis was used to determine the expression levels of M1 polarization-related proteins (IL-6, iNOS, TNF-α, and IL-1β) and M2 polarization-related proteins (Arg-1, CD206, and IL-10), as well as TLR-4/MAPK signaling pathway-related proteins. As the NET concentration increased, the M1 polarization effect of Kupffer cells gradually increased and the M2 polarization effect of Kupffer cells gradually decreased (**Figure 8A**), and the TLR-4/MAPK signaling pathway was significantly activated (**Figure 8B**). To elucidate the role of NETs in Kupffer cell M1 polarization and HMGB1 intracellular translocation, Kupffer cells were treated with DNase-1, a known NET inhibitor. We found that NETs can promote Kupffer cell M1 polarization by activating the TLR-4/MAPK signaling pathway and that DNase-1 partly reversed these effects (**Figures 8C–E**). Additionally, we discovered that NETs can stimulate HMGB1 translocation from the nucleus to the cytoplasm. Then, we determined whether suppression affects HMGB1 translocation. Assessment of the separated cytoplasmic and nuclear extracts revealed that the HMGB1 intracellular shift was suppressed by DNase-1 in Kupffer cells (**Figure 8F**).

### Co-Adjustment of Toll-Like Receptor 4 and mTOR Signaling Pathway Determines the Outcomes of Acute Rejection in Liver Transplantation

**Figure 9:**
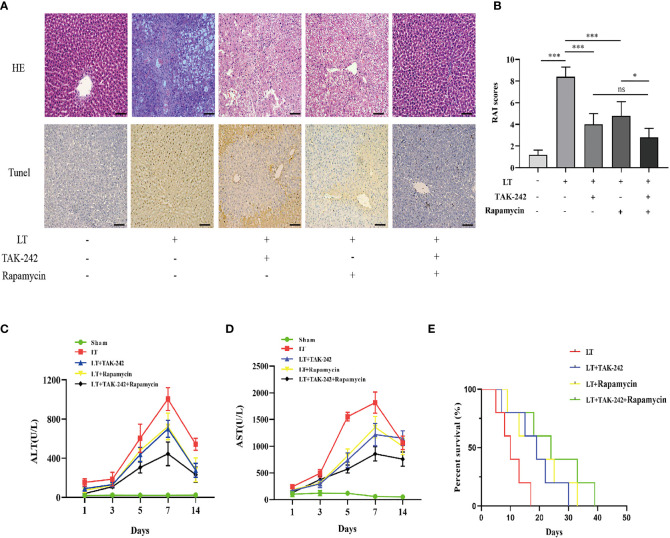
Effects of co-treatment of TAK-242 and rapamycin on AR following liver transplantation in rats. **(A)** Pathological changes in the liver and hepatic apoptosis following co-treatment with TAK-242 and rapamycin during AR (magnification, ×200; scale bar = 100 μm). **(B)** RAI was classified based on Banff patterns. **(C, D)** Serum levels of ALT and AST on days 1, 3, 5, 7, and 14 after liver transplantation. **(E)** Survival rate of rats. n = 6 for each group. Statistical differences among groups were assessed by one-way ANOVA. ^ns^ *P* > 0.05, ^*^ *P* < 0.05, ^***^ *P* < 0.001.

HE staining was used to determine the effects of TAK-242 (a TLR-4 inhibitor) and rapamycin (an mTOR inhibitor) on AR rats following liver transplantation. TAK-242 or rapamycin can both partially alleviate the severity of liver injury (**Figures 9A, B**). Consistent with previous research, TAK-242 or rapamycin treatment reduced apoptosis in liver tissue and hepatic aminotransferase levels in serum (**Figures 9A, C, D**). Following liver transplantation, the TAK-242+rapamycin treatment group exhibited better survival outcomes (**Figure 9E**). These findings suggested that combining TAK-242 and rapamycin could provide a more robust alleviation of AR following liver transplantation than either treatment alone.

## Discussion

Liver transplantation is the only effective therapeutic option for end-stage liver disease (22). Postoperative acute rejection (AR) in liver transplantation is the leading cause of graft dysfunction as well as loss (2, 23). Therefore, elucidation of the mechanisms involved in AR development following liver transplantation will inform the development of new therapies as well as treatment strategies. Our previous study proved that excessive neutrophil accumulation or hyper-responsiveness of neutrophils and an uncontrolled NET formation process after liver transplantation highly correlated with the formation of the local liver inflammatory microenvironment and AR following liver transplantation (13, 24). High-mobility group box 1 (HMGB1) is a damage-related molecular pattern molecule triggering the activation of macrophages in inflammatory microenvironments, resulting in tissue injury (25, 26). Moreover, numerous reports have revealed that HMGB1 activates NET formation by stimulating the HMGB1/TLR-4 axis in neutrophils (18, 27, 28). However, the mechanism by which HMGB1 promotes NET formation and NETs induce Kupffer cell M1 polarization and whether NET formation contributes to the pathogenesis of AR post-liver transplantation have not been elucidated.

We discovered that NETs were elevated in the serum of patients undergoing liver transplantation. NET levels decreased progressively throughout recovery and then stabilized. It has been reported that NET formation is associated with AR following liver transplantation. Thus, we determined the expression levels of serum NETs in AR in liver transplantation patients. Circulating levels of NETs were positively correlated with the expression of indictors of liver function in patients undergoing liver transplantation. Therefore, there may be a correlation between AR course and NET formation.

We conducted *in vitro* experiments to confirm the relationship between liver transplantation and NET formation and the ability of HMGB1 to promote NET formation based on serological findings. We discovered that neutrophils from liver transplant individuals can promote spontaneous NET formation. Additionally, HMGB1 stimulated NET formation in neutrophils isolated from healthy individuals. Therefore, liver transplantation and HMGB1 affect NET formation.

**Figure 10:**
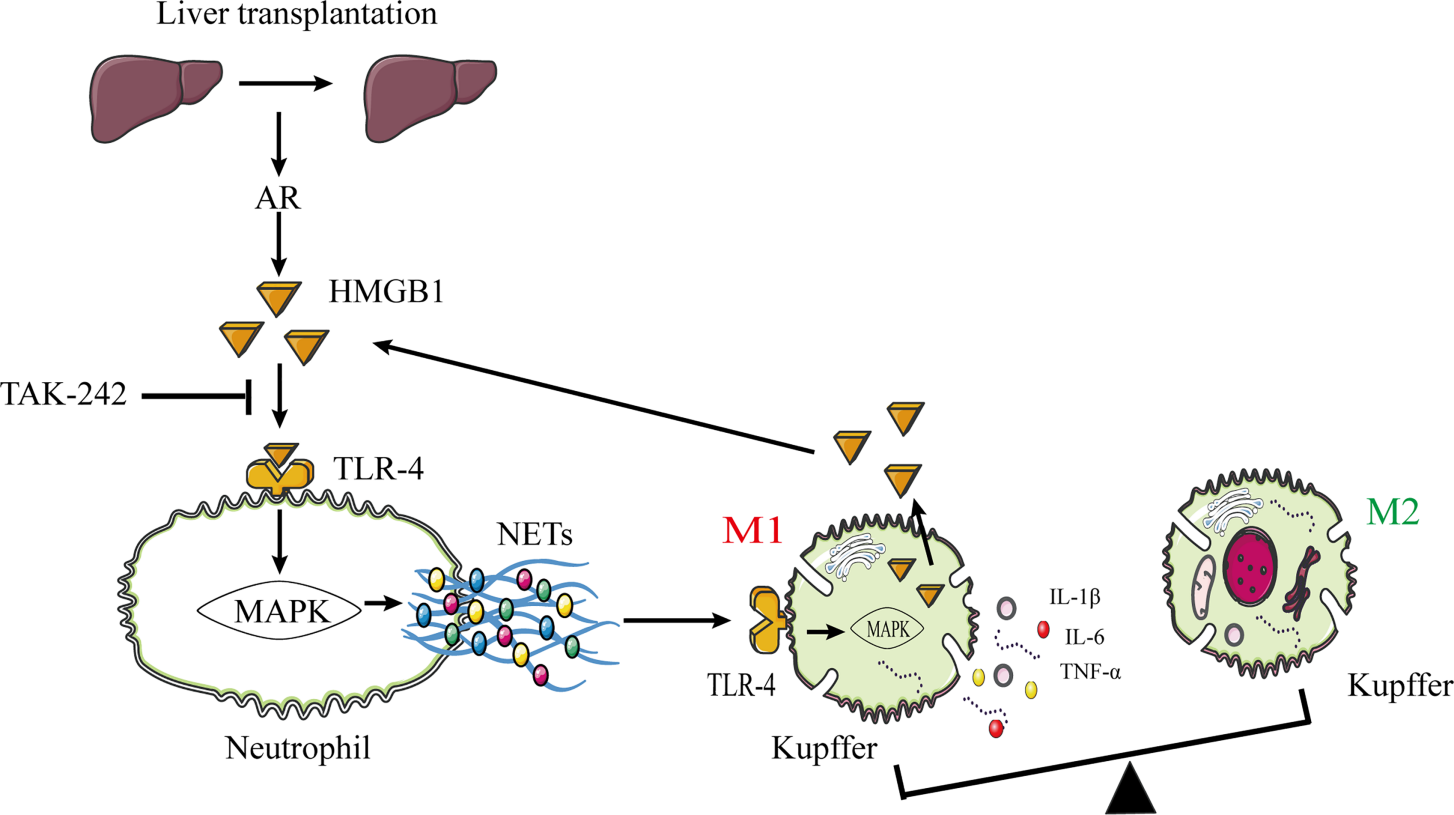
The proposed model shows that NETs induce M1 polarization of Kupffer cells and HMGB1 translocation *via* HMGB1/TLR-4/MAPK signaling pathway during AR following liver transplantation. HMGB1 promotes NET formation by activating the HMGB1/TLR-4/MAPK signaling pathway. NETs stimulate M1 polarization of Kupffer cells and HMGB1 intracellular translocation. Subsequently, HMGB1 is released into the extracellular milieu to aggravate liver injury. HMGB1, high-mobility group box 1 protein; TLR-4, toll-like receptor 4; MAPK, mitogen-activated protein kinase; AR, acute rejection.

Previously published studies reported that HMGB1 promotes NET formation *via* its interaction with TLR-4 (29, 30). Therefore, we confirmed that these changes were associated with the HMGB1/TLR-4/MAPK signaling pathway activation in human neutrophils stimulated by HMGB1. Our data showed that HMGB1 activated the HMGB1/TLR-4/MAPK signaling pathway during HMGB1-induced NETosis. Additionally, TAK-242 (a TLR-4 inhibitor) decreased the activation of the HMGB1/TLR-4/MAPK signaling pathway, which was reversed by LPS (a TLR-4 agonist), indicating that inhibiting the HMGB1–TLR-4 interaction and HMGB1 secretion in a TLR-4-dependent manner are required for HMGB1-induced NET formation. Our data showed that blocking TLR-4 with TAK-242 was therapeutic in rats following liver transplantation; however, other potential TLR4 ligands could account for this effect, and more specific mechanisms require further research. Additionally, we found that Kupffer cells are the primary HMGB1-secreting cells in the liver following immunological liver injury. Our major findings are summarized in a concept diagram (**Figure 10**).

Furthermore, we investigated the association between NET and Kupffer cell activation. With increasing NET concentration, the M1 polarization effect of Kupffer cells was gradually increased, and the TLR-4/MAPK signaling pathway was significantly activated. Additionally, we found that NETs can promote HMGB1 intracellular translocation in Kupffer cells, resulting in HMGB1 release into the extracellular milieu. Massive extracellular HMGB1 accumulation promotes NET formation in neutrophils. Eventually, interactions between neutrophils and Kupffer cells within the context of immune-mediated liver injury form a positive feedback loop that exacerbates liver damage. The underlying mechanism may be associated with the role of NETs in M1 polarization of Kupffer cells, HMGB1 intracellular translocation, and proinflammatory cytokine production *via* TLR-4/MAPK activation.

Numerous studies have established that activation of the mTOR signaling pathway is essential for Kupffer cell M1 polarization (31, 32). Rapamycin, as a well-known mTOR inhibitor, is one of the main immunosuppressive drugs used to prevent liver transplant rejection (33–35). However, the use of immunosuppressive agents is frequently associated with a plethora of side effects (36, 37). Numerous studies have discovered that treating AR following liver transplantation using a multi-target and multi-pathway approach may be a novel therapeutic strategy. We found that combining TAK-242 and rapamycin could more significantly alleviate AR following liver transplantation more effectively than either treatment alone.

In conclusion, NETs contribute independently to AR following liver transplantation, and the precise mechanism by which HMGB1 induces NET formation is associated with activation of the HMGB1/TLR-4/MAPK signaling pathway. Our findings revealed that NETs promote Kupffer cell M1 polarization and intracellular translocation of HMGB1, aggravating AR following liver transplantation. Additionally, the combination of TAK-242 with rapamycin may be a novel therapeutic strategy for AR following liver transplantation.

## Data Availability Statement

The original contributions presented in the study are included in the article/**Supplementary Material**. Further inquiries can be directed to the corresponding author.

## Ethics Statement

The studies involving human participants were reviewed and approved by the Ethics Committee of Animal and Human Experimentation of Chongqing Medical University. The patients/participants provided their written informed consent to participate in this study. The animal study was reviewed and approved by the Ethics Committee of Animal and Human Experimentation of Chongqing Medical University.

## Author Contributions

ZW, YLiu, and XP participated in designing the experiments and editing the final draft of the article. YLuo, XP, XQ, JG, ZH, TM, and BZ participated in performing the studies. All authors contributed to the article and approved the submitted version.

## Funding

This work was supported by the National Natural Science Foundation of China (No.81873592 and No.82170666).

## Conflict of Interest

The authors declare that the research was conducted in the absence of any commercial or financial relationships that could be construed as a potential conflict of interest.

## Publisher’s Note

All claims expressed in this article are solely those of the authors and do not necessarily represent those of their affiliated organizations, or those of the publisher, the editors and the reviewers. Any product that may be evaluated in this article, or claim that may be made by its manufacturer, is not guaranteed or endorsed by the publisher.

## Supplementary Material

Supplementary Figure 1 Immunofluorescence of neutrophils and kupffer cells. **(A)** Images of neutrophils isolated from patients (magnification, x200; scale bar=100μm). **(B)** Images of kupffer cells (F4/80+) isolated from rats (magnification, x400; scale bar=50μm).
