# Supplementary figures and images for: Neutrophil Extracellular Traps Regulate HMGB1 Translocation and Kupffer Cell M1 Polarization During Acute Liver Transplantation Rejection
Source: Front Immunol. 2022 May 6;13:823511. doi: 10.3389/fimmu.2022.823511 (PMC9120840; doi:10.3389/fimmu.2022.823511)

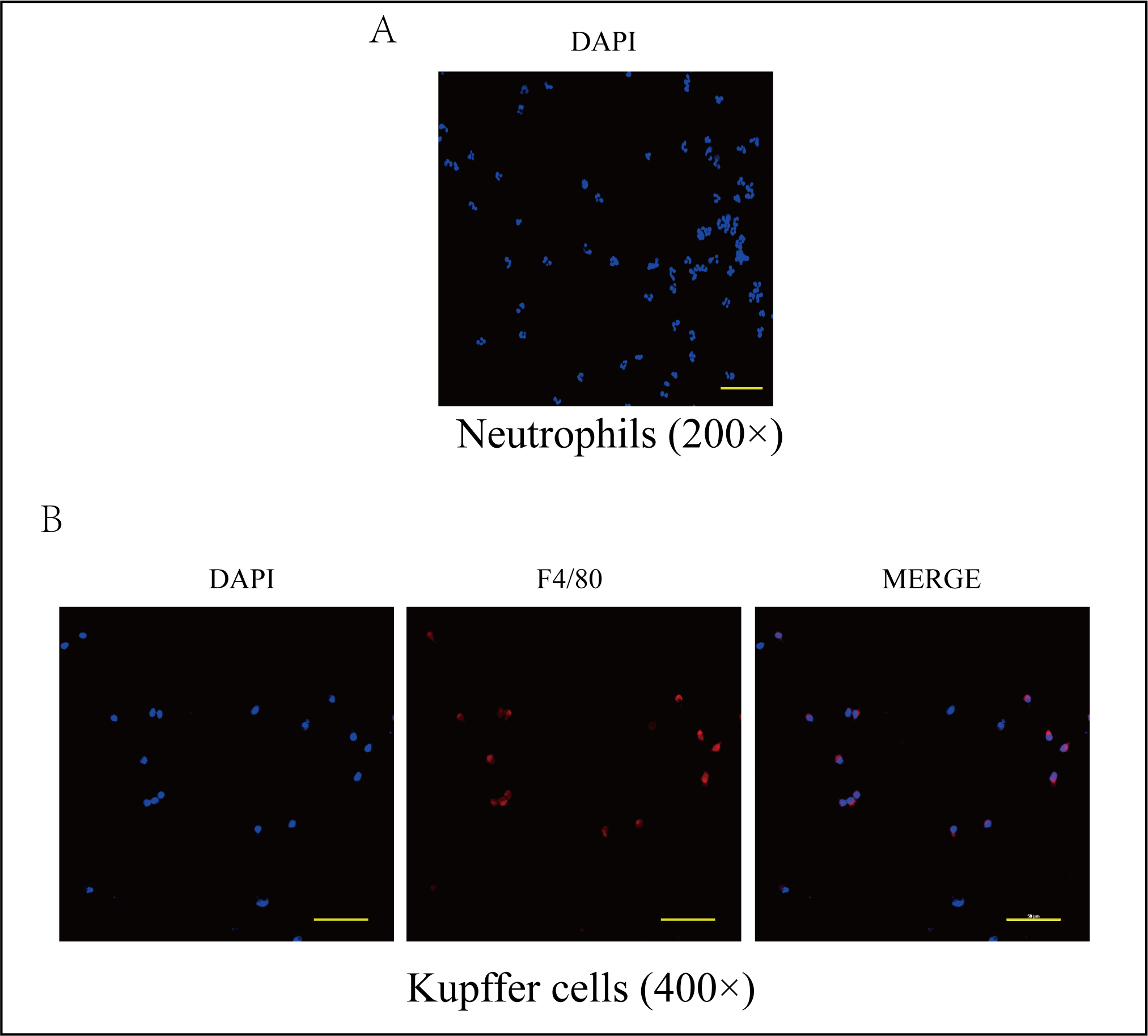

Supplement: Supplementary Figure 1 — Immunofluorescence of neutrophils and kupffer cells. (A) Images of neutrophils isolated from patients (magnification, x200; scale bar=100μm). (B) Images of kupffer cells (F4/80+) isolated from rats (magnification, x400; scale bar=50μm). [file Image_1.tif]
